# Neurological Involvement in Primary Sjögren Syndrome: A Focus on Central Nervous System

**DOI:** 10.1371/journal.pone.0084605

**Published:** 2014-01-20

**Authors:** Manuela Morreale, Pasquale Marchione, Patrizia Giacomini, Simona Pontecorvo, Massimo Marianetti, Claudio Vento, Emanuele Tinelli, Ada Francia

**Affiliations:** 1 Neuroimmunological Centre, Department of Neurology and Psychiatry, Sapienza - University of Rome, Rome, Italy; 2 Neurosonological Unit, Department of Medical and Surgical Sciences and Biotechnologies – Section of Neurology, Sapienza - University of Rome, Rome, Italy; 3 Department of Clinical Neurosciences, Neurological Centre of Latium - Institute of Neurosciences, Rome, Italy; 4 Neuropsychology Outpatients Service, Department of Medical and Surgical Sciences and Biotechnologies – Section of Neurology, Sapienza - University of Rome, Rome, Italy; 5 Neuroradiological Unit, Department of Neurology and Psychiatry, Sapienza - University of Rome, Rome, Italy; College of Mechatronics and Automation, National University of Defense Technology, China

## Abstract

**Objectives:**

Sjögren syndrome is an autoimmune disease involving mainly salivary and lacrimal glands. Beyond widely described PNS involvement, high variable prevalence of CNS manifestations ranging from 2.5 and 60% of all pSS patients has been reported, without specific syndrome definition. The aim of this cohort study was to evaluate the prevalence of CNS signs and symptoms in pSS patients and to identify possible biomarkers of CNS damage.

**Methods:**

120 patients with pSS diagnosis according to the 2002 American-European Consensus Group criteria were enrolled after exclusion of secondary causes. All patients underwent to a wide neurological, neuropsychological, psychiatric, neuroradiological and ultrasonographic evaluation.

**Results:**

Central and peripheral nervous system involvement was observed in 81 patients with a prevalence of 67.5%. The prevalence of CNS involvement was significantly higher than PNS disease (p 0.001). 68 patients (84%) shown non-focal CNS symptoms and 64 (79%) focal CNS deficits with headache as the most common feature (46.9%), followed by cognitive (44.4%) and mood disorders (38.3%). Particularly, we observed a high prevalence of migraine without aura, subcortical frontal executive functions and verbal memory impairment and apathy/alexythimia. MR spectroscopy revealed a reduction of NAA levels or NAA/Cr ratio decrease in subcortical frontal and basal ganglia white matter, while ultrasonography showed an impairment of microvasculature response. At multivariate analysis, headache, cognitive disorders and psychiatric symptoms was significantly associated to serological markers (anti-SSA), MRS and ultrasonographic features.

**Conclusions:**

The higher prevalence of MWO-mimic headache, cognitive dys-esecutive syndrome and mood disorders observed in this series confirmed previous evidences of a higher diffused CNS compromission rather than focal involvement such as SM-like clinical course or NMO-like syndrome. The association with immunological biomarkers, metabolic cerebral dysfunction and microvascular damage suggests a possible endothelial dysfunction of the cerebral microcirculation or a potential inflammation-mediated shift of the neurovascular coupling.

## Introduction (Background)

Sjögren’s syndrome (SS) is a chronic, autoimmune disease clinically characterized by typical dryness of the mouth and eyes associated to involvement of other exocrine glands as well as a wide variety of organs and systems [1]. Beyond the primary disease (pSS), an association with other autoimmune rheumatic diseases has been observed and defined as secondary Sjögren’s syndrome (sSS). Mononuclear cell infiltrate and progressive injury of the exocrine glands are the main pathological features of the disease. Despite a prevalence ranging between 1% and 3% of the general population, more than 50% of patients has not received a correct diagnosis and approximately 30% of patients presenting other autoimmune diseases suffered from pSS [1], [2]. Female gender was more affected with a F:M ratio of about 9∶1 in the age group between 40 and 50 years [3]–[5]. Since Peripheral Nervous System (PNS) involvement has been widely described from several epidemiological, pathophysiological and histopathological studies, Central Nervous System (CNS) manifestations are still object of debate. The first observation of the CNS involvement with focal or diffuse symptoms is the series of 8 patients described by Alexander et al. in 1982, in which a direct etiopathogenetic role of the anti-Ro (SSA) antibodies was suggested [6]. Although severe neuropsychiatric syndromes may occur even in the seronegative forms of pSS, some Authors suggested the importance for a neurologist to be familiar with the extraglandular manifestations of this syndrome [7], [8]. After the introduction of the current diagnostic criteria for the Sjögren's syndrome in 2002 [2], an increased and high variable prevalence ranging from 2.5 and 60% of CNS sign and symptoms has been observed. The lack of syndromic definition of CNS involvement in pSS may determine a selection bias in the studies that may explain the wide prevalence data, particularly for initial impairment of one or more systems such as Mild Cognitive Impairment (MCI) [9], [10]. Furthermore, CNS involvement may precede clinical diagnosis by many years and determine an underestimation of other neurological and/or systemic diseases [11]. The aim of this cohort study was to evaluate the prevalence of CNS signs and symptoms in a population of consecutive patients with pSS and to identify possible biomarkers of subclinical and clinical CNS damage by means of a wide diagnostic work-up based on clinical and instrumental evaluation with functional and structural neuroimaging, neurosonology and neuropsychology.

## Results

120 patients affected with pSS were included in the study. As expected, large amount of patients were female with a M:F ratio of 1 to 9 (12 males and 108 females; mean age 58.3±14.2 years). Central and peripheral nervous system involvement was observed in 81 patients (4 M – 4.9% and 77 F – 95.1%) with a prevalence of 67.5%. The clinical-demographic characteristics of the patient group with neurological involvement are shown in Table 1. According to clinical history, neurological onset was estimated over an average period of 16.7±6.2 years, while mean time from immunological diagnosis was 9.3±6.8 years, with an average time difference of 7.4±5.6 years. In 82.7% (n  =  67) of the patients, anti-SSA and anti-SSB was positive.

**Table 1:** Clinical-demographic characteristics with pSS and neurological involvement.

Total patients with neurological symptoms (%)	n. 81 (67.5%)
female sex (%)	n. 77 (95%)
average age (years) ± ds	54.5±11.6
disease duration (years) ± ds	9.3±6.8
neurological onset - first symptom (years) ± ds	16.7±6.2

**Figure 1:**
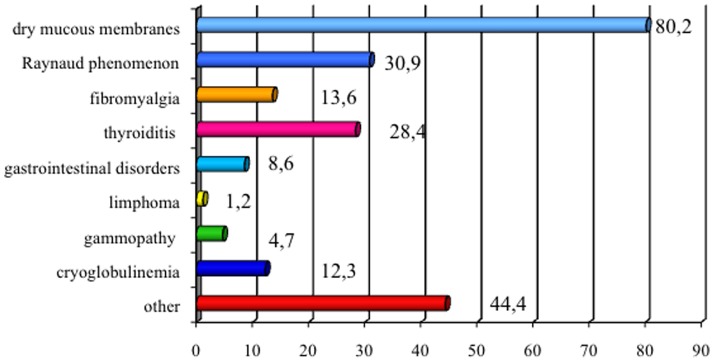
Prevalence of Systemic Symptoms in pSS Patients with Neurological Symptoms (%).

**Figure 2:**
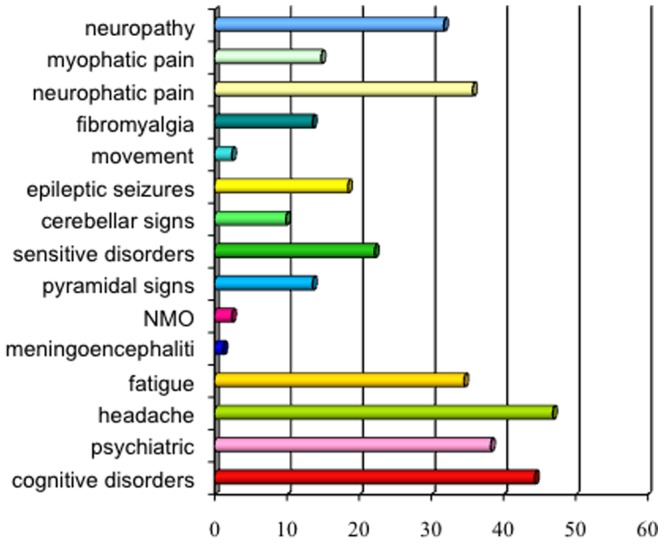
Neurological Involvement in the Course of pSS (%).

**Figure 3:**
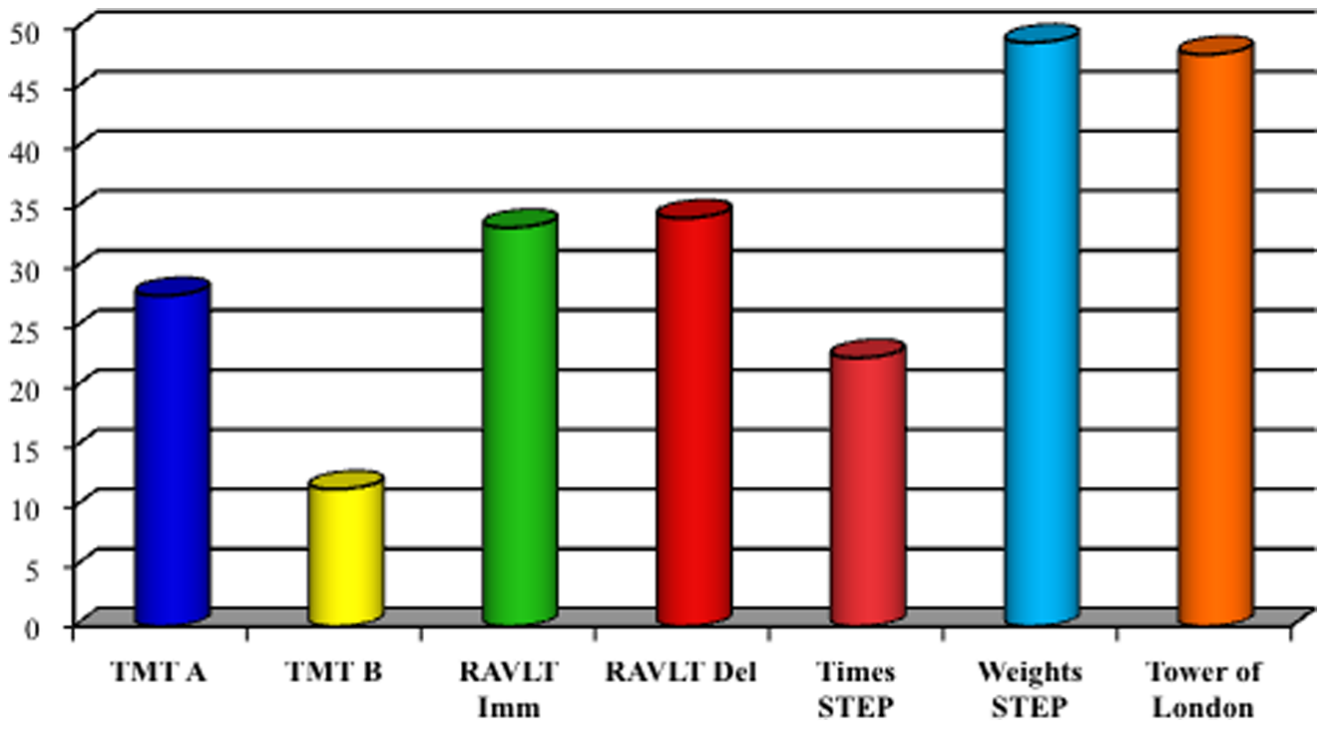
Neuropsychological Scores (%).

**Figure 4:**
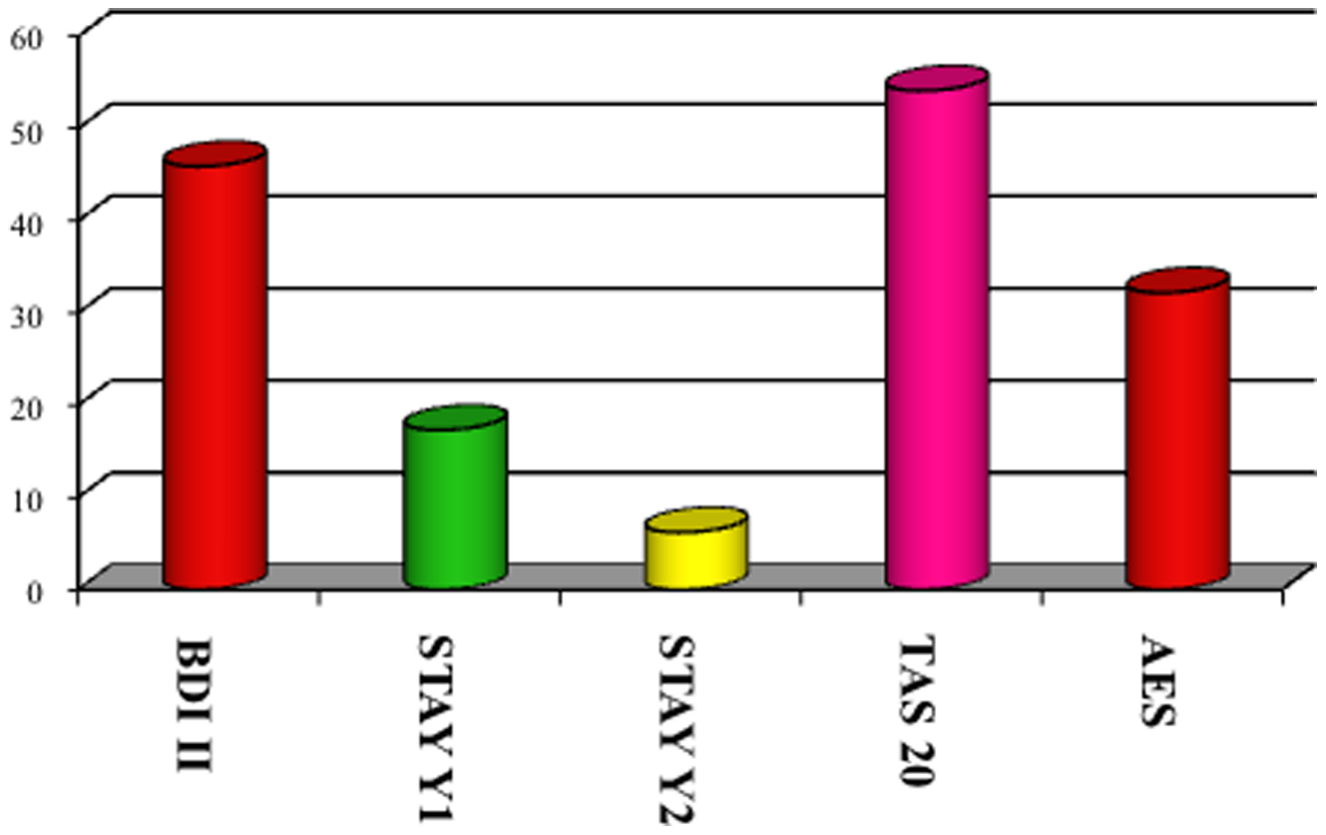
Neuropsychiatric Scores (%).

Particularly, ENA/SSA were present in 68 (84%) patients, ENA/SSB in 22 (27.2%) and ANA in 39 (48.1%) (p 0.000 and p 0.001, respectively). Gland biopsy confirmed pSS diagnosis in the remaining seronegative cases (17.3%, n  =  14). The prevalence of both systemic symptoms and comorbidities in patients with neurological involvement is shown in Figure 1. "Other" category included most rarely symptoms of pSS, such as joint pain and arthritis, skin disorders, bronchopulmonary disease and other disorders of the genitourinary apparatus. According to clinical history and neurological examination, 68 patients (84%) shown non-focal neurological symptoms and 64 (79%) focal neurological deficits. In 43 (53%) of the cases, signs and symptoms of a PNS disorder were observed. The prevalence of CNS involvement was significantly higher than PNS disease (p 0.001), as well as non-focal neurological involvement was significantly greater than the focal (p 0.005). The distribution of CNS symptoms is shown in Figure 2. Headache was the most common neurological symptom (46.9%), followed by cognitive disorders (44.4%) and mood disorders (38.3%). Headache was defined on the basis of the ICHD-II diagnostic criteria and their relative frequencies are shown in Table 2. The most frequently observed headache satisfied ICHD-II criteria for migraine without aura (MWO) with generally unilateral pain, oppressive-throbbing type and moderate-to-severe intensity, worsened by physical activity and associated with nausea, photophobia and phonophobia. In 25 (31%) patients with headache, head pain was associated to cutaneous allodynia, particularly in MWO cases (t 3.4, df 79, p 0.001). The MWO was significantly more frequent in patients with positive antibodies SSA (t 3.25, df 79, p 0.002), MRS pathological features (t 7.49, df 67, p 0.000) and Raynaud's phenomenon (t 2.163, df 79, p 0.02). From the cognitive point of view, patients obtained proper scores in line with standard examinations for all tests except for the Tower of London, Trail Making Test A and B, Times and Weights STEP and Rey Auditory Verbal Learning Test, as involvement of subcortical frontal executive functions and verbal memory (Figure 3). During the execution of the Tower of London, the most frequent error was the breach of the rules rather than a lack of understanding of the task, in relation to a phenomenon of cognitive perseveration. The alteration of the STEP weights and measures, compatible with a deficit of decision making is significantly correlated with the alteration on the compulsive shopping scale (ρ 0.314, p 0.004). The scores obtained at the Tower of London, TMT A and B and STEP are significantly correlated with those obtained in the RAVLT (respectively, ρ 0.429 p 0.000, ρ 0.308 p 0.002, ρ 0.346 p 0.004, ρ 0.314 p 0.004), in relation to a disturbance of verbal memory as an expression of subcortical frontal dysfunction. The results of the neuropsychiatric evaluation are shown in figure 4. 45.8% of patients scored higher than 14 on the BDI-II as in depressive disorder, while STAY Y1 and Y2 were pathological in 17.3% and 6.2% respectively. At TAS-20, 54.1% of patients obtained a score higher than 61, in line with high-grade alexithymic disorder and a further 21% of cases obtained a score between 51 and 61, in agreement with mild alexythimia. A high score on the apathy evaluation scale (AES) was observed in 32.2%. A significant positive correlation was present between the BDI-II score and the AES (ρ 0.4, p 0.001), and between the TAS-20 score and the AES (ρ 0.57, p<0.001). Ultrasound evaluation shows a mean MIT value of 1.1±0.2 mm with a percentage of 39.2% patients having a value exceeding 0.9 mm. Stenosis lower than 30% at carotid level was found in 29 patients (35.3%), while a reduction of the lumen between 30% and 70% were found in 15 (18.3%) patients. No cases of high-grade stenosis and occlusion of the carotid artery or atherosclerotic disease in the vertebro-basilar system were observed. Intracranial stenosis at the level of middle cerebral artery (MCA) was observed only in 1 patient (1.2%). Independently of cardiovascular risk factors, mean pulsatility index (PI 1.3±0.6, nv 0.9±0.6, p<0.02), S/D ratio in the anterior circulation (3.4±1.7, nv 1.4±0.5, p 0.001) and breath holding index (BHI) at the MCA (1.8±0.1, nv 0.78±0.3, p <0.001) were found above the reference values for the laboratory. No changes in the S/D ratio and BHI were in the posterior circulation. Structural brain damage such as lacunar infarction or MS-like lesions at MRI was present in 42/81 patients (51.9%). A reduction of NAA levels or NAA/Cr ratio decrease in subcortical frontal and basal ganglia white matter at MRS was present in 47/81 (58%). Both alterations were present in 24/81 (29.6%). The presence of a significant MIT and/or atherosclerotic plaques was not associated with the presence of both lacunar infarcts and MS-like lesions or metabolic damage at MRI (p>0.05). On the contrary, the increase of the S/D ratio and BHI on the anterior circulation was positively and significantly related to the MRS alteration (ρ 0.85, p<0.001) but not with structural damage (ρ – 0.15, p 0.082). The results of multivariate analysis (MANOVA) of the neurological symptoms in relation to sero-instrumental markers are reported in Tables 3 and 4. The presence of SSA significantly increased the risk of headache (OR 2.85, 90% IC 1.43–9.6; p 0.001), mood disorders (OR: 2.72, 90% IC 2.95–13; p<0.001) and fibromyalgia (OR: 1.99, 90% IC 1.21–12.8; p 0.001). The reduction of NAA levels or NAA/Cr ratio decrease at NMR twicely increase the risk of both headache (OR: 2.76, 90%IC 0.6–11.45; p 0.005) and mood disorders (OR: 1.98, 90% IC 1.15–7.3; p 0.007). On the other hand, higher S/D ratio on MCA and BHI are related to increased risk of headache (OR: 2.73, 90% IC 1.21–12.6, p 0.001 and OR: 2.76, 90%IC 0.6–11.45, p 0.005 respectively) and cognitive disorders (OR: 2.46, 90% IC 1.05–8.7, p 0.001 and OR: 1.98, 90% IC 0.73–8.4, p 0.002 respectively). Particularly, an increased BHI significantly predict the NAA levels or NAA/Cr ratio decrease at NMR (OR: 2.56, 90% IC 1.23–9.8; p 0.001).

**Table 2:** Clinical subtypes of headache in pSS patients.

	N (%)
MWO	39 (48.1%)
MWA	1 (1.2%)
CM	5 (6.1%)
eTTH	17 (21%)
cTTH	11 (13.8%)
MOH	8 (9.8%)

Legend: migraine without aura - MWO (Migraine without aura), migraine with aura - MWA (Migraine With Aura), chronic migraine - CM (Chronic Migraine), episodic tension-type headache - eTTH (episodic Tension-Type Headache), headache chronic tension-type headache - CTTH (chronic Tension-Type headache); analgesic overuse headache - MOH (Medication overuse headache).

**Table 3:** Multivariate analysis: neurological symptoms and serologic-instrumental markers.

	anti SSA	OR	MRS	OR
**mood disorder**	<0.001	2.72 (90% IC 2.95–13)	0.007	1.98 (90% IC 1.15–7.3)
**cognitive disorders**	0.02	1.81 (90% IC 1.12–9.8)	0.02	1.88 (90% IC 0.9–11.4)
**headache**	0.001	2,85 (90% IC 1.43–9.6)	0.005	2.76 (IC 0.6–11.45)
**fatigue**	0.02	1.84 (90% IC 1.2–16.8)	-	-
**cerebellar**	-	-	-	-
**pyramidal**	-	-	-	-
**fibromyalgia**	0.001	1.99 (90% IC 1.21–12.8)	-	-
**neurophatic pain**	0.02	1.87 (90% IC 1.1–9.8)	-	-

**Table 4:** Multivariate analysis: neurological symptoms and serologic-instrumental markers.

	S/D ratio	OR	BHI	OR
**mood disorder**	0.01	1.88 (90% IC 0.95–5.9)	-	-
**cognitive disorders**	0.001	2.46 (90% IC 1.05–8.7)	0.002	1.98 (90% IC 0.73–8.4)
**headache**	0.001	2.73 (90% IC 1.21–12.6)	0.005	2.76 (IC 0.6–11.45)
**fatigue**	-	-	-	-
**cerebellar**	-	-	-	-
**pyramidal**	0.02	1.79 (90% IC 1.12–14.8)	-	-
**fibromyalgia**	-	-	-	-
**neurophatic pain**	-	-	-	-
**MRS**	0.01	1.83 (90% IC 1.36–11.8)	0.001	2.56 (90% IC 1.23–9.8)

## Discussion

The involvement of CNS and PNS in autoimmune diseases is one of the most emerging and debated topic because of poor standardized studies. Although many data about the incidence and the aetiology of peripheral neuropathy during pSS have been published [34], only in the last decades the increased relevance of CNS symptoms on pSS arises from literature. More specific diagnostic criteria for pSS and a wide definition of the neurological complications may account for the differences among the earliest and the most recent studies [35]–[38]. Particularly, diffuse non-focal neurological signs and symptoms are considered more prevalent. Escudero et al. recently observed a significant prevalence of headache as an expression of radiological subclinical involvement of the SNC in pSS, but the clinical characteristics of pain remain undefined [39]. Moreover, Lafitte et al. described a frontal cognitive dysfunction that was not apparently related to a structural damage at neuroimaging [40], while Malinow et al. reported a significant psychiatric impairment in about half of the examined cases [41]. According to Belin et al., neuropsychiatric dysfunction fails to correlate with structural damage and seems to be related to "functional" neurological abnormalities at SPECT [42]. The higher prevalence of headache, cognitive dysfunction, mood disorders and fatigue observed in this series confirmed previous evidences of a higher diffused CNS compromission rather than focal involvement such as SM-like clinical course or NMO-like syndrome [9], [43], [44]. In order to define clinical features of headache, diagnostic criteria of migraine without aura (MWO) were more frequently applicable, while tensive subtypes and chronic or medication overuse complications are less frequent. Independently from age and sex, MWO are significantly related to SSA antibodies, MRS alterations and haemodynamic dysfunction at ultrasonographic evaluation but not to the presence of vasculitic brain lesions and/or macrovascular damage. In addition, the frequency of headache and alterations to MRS is higher in patients with Raynaud's phenomenon. These results suggested a possible endothelial dysfunction of the cerebral microcirculation or a potential inflammation-mediated shift of the neurovascular coupling and excluded a microangiopathic or thrombotic mechanism, as in antiphospholipid syndrome. Thereby, headache in pSS may be related to an "autoimmune endotheliitis" that directly alters the biochemical and humoral milieu, consequently inducing perivascular inflammation and a vasomotor dysfunction. According to Escudero et al, migraine-mimic headache during pSS could be a direct expression of the disease and not a mere comorbidity, as suggested in the NP-SLE [39]. Furthermore, dysfunction of rexecutive functions seems to characterize cognitive deficits in pSS, as expression of such an impairment of the frontal-subcortical circuits, while primitive memory impairments were not observed. Particularly, violations of the rules by a mechanism of perseveration and a high number of errors during task performance were observed in a high percentage of patients, clearly accounting for a dys-executive syndrome (verbal memory). The inability of pSS patients to produce a cognitive assessment and to take appropriate decisions partially correlates to problematic shopping scores. The lack of specific structural changes at MRI suggests a possible premotor cortex involvement rather than mesolimbic cortical impairment, but this hypothesis needs further confirmations. Interestingly, cognitive dysfunctions are significantly related to the presence of SSA and MRS changes, independently from age and sex. Therefore, despite late onset of pSS, cognitive symptoms are more prevalent than age-related dysfunction and could be attributable to a subclinical inflammatory damage and not to structural microvascular damage (white matter lesions), as in cerebrovascular disease or in migraine [45]. On psychiatric point of view, our study found a significant prevalence of alexythimia, apathy and depression in pSS patients. Alexythimia is characterized by difficulty in recognizing and identifying emotions, dysfunction in distinguishing emotions from somatosensory feelings and by a particular cognitive attitude to concrete experiences (46). Some studies on small cohorts have found a higher prevalence of alexythimia in patients with autoimmune diseases (RA, SLE) [46]. Recently, Van Leeuwen et al. observed that patients with pSS not differ from controls in terms of the processing and regulation of emotions, but rather in terms of interpretation and expression of the same feelings in accordance with the definition of alexythimia [47]. Moreover, neuroimaging studies have observed a correlation between the alexythimia construct and the cortico-subcortical frontal-temporal structures, engaged both in the processing of emotions and in executive functions [46]. As criticism, pSS is a late-onset chronic disease in which reactive symptoms such as emotional lability, masked depression and emotional withdrawal could mislead alexythimia. Effectively, pSS patients appear detached, sometimes anhedonic and weak from the emotional point of view during the psychiatric interview. However, we found a dysfunction in the specific ability to understand and decode the emotions and the emotional means. Out of a mere psychological discomfort, the correlation among alexythimia, apathy and depression suggest an organic substrate and a possible correlation with cognitive dysfunctions. According to this model, depressive scores significantly correlate to SSA antibodies, MRS alterations and cerebrovascular dysfunction in our series. The lack of association among alexythimia, apathy, autoantibody profile and neuroimaging is maybe due to small sample and the cross-sectional design of the study. An object of debate is which was the best neuroimaging of CNS involvement in pSS and generally in autoimmune diseases both in the diagnostic and in the follow up stage. Although SPECT was considered more sensitive in previous studies, we observed a specific alteration of metabolic markers tissue at MRS in front of less frequent presence of structural damage. MRS provides functional information about the brain region in which they are examined and have been frequently used in other neuroimmunological conditions such as neuropsychiatric Systemic Lupus Erythematosus [48]. Probably, the quite non-specific and diffuse neurological symptoms could be related to an early subtle and functional impairment of CNS due to supposed endotheliitis. Effectively, reduction of NAA levels and NAA/Cr ratio decrease in subcortical frontal and basal ganglia white matter at MRS are significantly related to ultrasonographic impairment of microvasculature. According to our results, MRS impairment may be an early bio-marker of CNS involvement in pSS which result less expensive and invasive than other functional imaging. Despite the above-mentioned limitations of this study, the concept of Neuro-Sjögren seems reasonable in the same fashion of NP-SLE [49]. Non focal involvement of CNS is more frequent than focal neurological symptoms, suggesting clinical and pathological differences with macroscopic vasculitic damage (white matter lesions, MS-like), as occurring in other neuroimmunological diseases [50]. Moreover, neurological onset may often preexist both the appearance of systemic symptoms and the immunological diagnosis by many years. Thus, a pSS should always be taken into account in patients with relatively non-specific neurological symptoms, such as headaches or neuropsychiatric symptoms associated to sicca syndrome. MRS, ultrasound screening and neuropsychological assessment might represent effective for an early diagnosis, particularly when neurological symptoms precede systemic involvement.

## Materials and Methods

Between January 2010 and January 2013, all consecutive outpatients with pSS referred to Neuro-Immunology Center of the Neurology and Psychiatry Department – SCAN Onlus (Sapienza, University of Rome) were enrolled. All subjects voluntarily participated the study after written informed consent concerning treatment of personal data. Scientific/Ethic committee of SCAN Onlus approved the study. Since the observational design of this cohort study, no control subjects were enrolled. AECG criteria [2] were used for diagnosis of pSS by an expert rheumatologist. All secondary forms and undifferentiated connective tissues pathologies were considered as different disease from pSS. Further exclusion criteria was positive hepatitis C biomarkers that may be associated with pathological changes in minor salivary glands mimicking siccae syndromes [12]. Patients with cryoglobulinemia and Hashimoto's thyroiditis symptoms were not excluded since they do not represent nosographic entities per se.

### Clinical evaluation

All patients underwent a wide anamnestic investigation by means of ad hoc semistructured interview and a complete objective neurological examination. Disease duration, serological tests and the onset of neurological symptoms were taken into account. Specific subjective symptoms were evaluated differently with appropriate tool as presented in Table 5. The Second Edition of the International Classification of Headache Disorders (ICHD-II) was applied to perform the headache diagnosis [13].

**Table 5:** Clinic evaluation scales for subjective symptoms.

SYMPTOM	SCALES
headache	semi-structured interview and headache diary
fibromyalgia	tender point on physical evaluation
fatigue	FSS
psychiatric disorders	psychiatric interview and CGI
depression	BDI
cognitive impairment	MMSE, TIB
pain	VAS, DN4
pyramidal symptoms	mRS

### Serology

Laboratory tests included: haemochrome, thyroid hormones, renal and hepatic function and inflammatory markers (ESR, C-reactive protein), autoantibody screening that included anti-nucleus (ANA), anti double strand-DNA (ds-DNA), anti extractable nuclear antigens (ENA) and central and peripheral anti-neutrophil cytoplasmic antibodies. ENA screening included anti-Ro/SSA, anti-La/SSB, anti-RNP, anti-SM, anti-jo1, anti-Scl70. Cryoglobulinemia and antineuronal antibodies were also evaluated.

### Ultrasonographic evaluation

The ultrasound examination has been performed by General Electric Logiq Pro echographic equipment with 7–11 MHz probe for extracranial vessels and 2.5–5 MHz probe for intracranial arteries. Carotid and vertebro-basilar systems were studied by means of B-mode evaluation in Color and Power Doppler and Pulsed-Wave technique for mean flow velocities assessment (predetermined depth, Pulse Repetition Frequency (PRF) <1, angle of insonation <60°). According to the criteria of the NASCET study [14], the thickness of the myointimal complex was measured around the distal area of the common carotid artery at about 1 cm from the carotid bifurcation by a software-included caliper, taking into account the mean value of three repeated measurements. By means of the same criteria [14], the minimum value of the amplitude for the definition of the atherosclerotic plaque was 1.4 mm and the percentage of stenosis was calculated as the proportion between the residue calibre in the point of maximum stenosis and the diameter of the downstream area. Further velocimetric evaluation was performed according to the instructions of the Consensus Conference of the Society of Radiologists in Ultrasound [15]. Intracranial arteries were insonated trough transtemporal bone window for the anterior circulation and suboccipital bone window for the vertebrobasilar circulation with a 2.5–5.5 MHz probe. B-mode/Color-Doppler methods were used for anatomic imaging of the vessels and Pulsed-Wave for the sound identification. Intracranial stenosis were diagnosed on the basis of the presence of a flow artifact in Color Doppler (aliasing fix type) and focal increments of the flow velocities, according to sub-mentioned Consensus Conference. Furthermore, all patients were evaluated by means of a blinded transcranial assessment with a 2–4 MHz probe, MultiDopX (Compumedics Inc.), in order to record the mean velocity flow (MVF), the pulsatility index (PI), the systolic-diastolic ratio (SDR) and the cerebrovascular reactivity. Breath-hold with short apnea without Valsalva Manoeuvre was used to test cerebrovascular reactivity (Breath Holding Index, BHI) as previously described [16].

### Neuroimaging

A standard Magnetic Resonance Imaging (MRI) examination was performed using a Philips Gyroscan NT Intera device at 1.5 Tesla with sequences in the axial plane: T1-weighted (TR: 582 ms, TE: 15 ms, slice thickness/gap: 5/1 mm), dual weighted imaging (TR: 2800 ms, TE: 110/20 ms, slice thickness/gap: 5/1 mm), T2-FLAIR technique (TR: 6000 ms, TE: 100 ms, TI: 2000 ms, slice thickness/gap: 5/1 mm) and sequences weighted in Diffusion (TR: 6000 ms, TE: 95 ms, slice thickness/gap: 5/1 mm, b  =  0–1000) in order to highlight a possible structural damage of the white matter as lacunar infarctions or MS-like lesions. Maps of apparent diffusion coefficient (ADC) were also created. The results were expressed as dichotomised variable with 0 – no lesions and 1 – lesions, independently from lesion load. We also performed a multivoxel H-Magnetic Resonance Spectroscopy (MRS) study with PRESS (Point Resolved Spectro-Scopy) sequence for subtle metabolic damage. The PRESS sequence allows the spectra acquisition with the higher signal-to-noise ratio, using a sequence with predominantly long TE, that has the privilege to produce data with double signal-to-noise rate, but has the disadvantage of being technically limited down in the choice of the echo time. This sequence is spin echo type with a second pulse at 180° responsible for the signal that will be acquired (second echo). The water suppression was achieved using three selective pulses of saturation with Chemical-Shift technique (CHESS). The volume of interest (VOI) was placed in all patients examined using the following parameters: TR – 1500 ms, TE – 144ms, matrix 16, data points 1024, FOV – 160×160 mm and nominal resolution in the transverse plane 1×1 cm. The choice of the sequences and that of analyzed metabolites was based on the protocols study reported in the literature. We analyzed voxel in correspondence of the frontal white matter, the posterior periventricular white matter and the basal ganglia bilaterally according to the hypothesis that a vasculitic damage of the white matter may underlie CNS involvement in pSS. The spectra were post processed using software supplied by the machine. The analyzed metabolites included the N-acetyl aspartate (NAA 1.9 to 2.1 ppm), the Choline (Cho 3.1 to 3.3 ppm), the Creatinine (CR 2.9 to 3.1 ppm), the Lipids and Macromolecules (Lip & Mac 1.2 to 1.4 ppm). The results were expressed both in terms of absolute amount of the substances under exam and in the ratio between the following metabolites: NAA/Cho and Cho/Cr.

### Psychiatric evaluation

The psychiatric assessment was based on anamnestic and clinical interview focused on: current or past psychopathological disorder; structure of the personality; signs and symptoms of reactive disorders to chronic illness, steroids or disease-modifying (biological) therapies. The diagnostic/nosological overview was obtained by following the nomenclature of the Diagnostic and Statistical Manual of Mental Disorders (DSM IV) [17]. Moreover, all patients underwent a global screening test with Clinical Global Impression Severity Scale (CGIs) and following specific tests to detect possible behavioural correlates of executive functions: Beck Depression Scale II – BDI-II [18], State-Trait Anxiety Inventory Y1 and Y2 – STAY [19], Toronto Alexythimic Scale - TAS-20 [20], Apathy Evaluation Scale – AES [21], Scale of problematic shopping [22].

### Neuropsychological assessment

Basal cognitive evaluation was based on Mini-Mental State Examination (MMSE) for cognitive efficiency and Brief Intelligence Test (TIB) for IQ [23], [24]. Regardless of screening tests, all patients underwent a complete neuropsychological assessment in order to explore the main cognitive domains by means of: Verbal Span [25]; Trail Making Test (parts A & B) [26]; Rey Auditory Verbal Learning Test [27]; The Rey-Osterrieth Complex Figure Test (ROCF) (with immediate and delayed recall) [28]; Test of Weights and Measures Estimation (STEP) [29]; Test of Phonological Verbal Fluency/Semantics [30]; Corsi Block Tapping Test [31]; Tower of London - Italian version [32]; Token Test and Aachener Aphasie Test [33]. Raw scores have been adjusted for age, sex, education parameters and where applicable, test-specific correction factors.

### Miscellaneous

Neurophysiological evaluation with electroencephalogram (EEG), evoked potentials (EP), electromyography (EMG) and electroneurography (ENG) were not considered mandatory and performed only in relation to the clinical picture.

### Statistical analysis

The prevalence of individual values was calculated through frequencies analysis and subsequently the distribution of the individual variables was evaluated. Then, a univariate analysis was performed with Student's t and Pearson χ^2^ test. To check the statistical significance between the clinical symptoms and the different markers of disease, the Multivariate ANalysis Of Variance (MANOVA) between groups and the Pearson correlation coefficient was performed. The level of significance was set at p<0.05 (confidence interval 95%). SPSS software, version 16.0 was used.
